# Physically Consistent Resolving Simulations of Turbulent Flows

**DOI:** 10.3390/e26121044

**Published:** 2024-11-30

**Authors:** Stefan Heinz

**Affiliations:** Department of Mathematics and Statistics, University of Wyoming, 1000 E. University Avenue, Laramie, WY 82071, USA; heinz@uwyo.edu

**Keywords:** computational fluid dynamics, large eddy simulation (LES), Reynolds-averaged Navier-Stokes (RANS) methods, hybrid RANS-LES methods

## Abstract

Usually applied simulation methods for turbulent flows as large eddy simulation (LES), wall-modeled LES (WMLES), and detached eddy simulation (DES) face significant challenges: they are characterized by improper resolution variations and essential practical simulation problems given by huge computational cost, imbalanced resolution transitions, and resolution mismatch. Alternative simulation methods are described here. By using an extremal entropy analysis, it is shown how minimal error simulation methods can be designed. It is shown that these methods can overcome the typical shortcomings of usually applied simulation methods. A crucial ingredient of this analysis is the identification of a mathematically implied general hybridization mechanism, which is missing in existing methods. Applications to several complex high Reynolds number flow simulations reveal essential performance, functionality, and computational cost advantages of minimal error simulation methods.

## 1. Introduction

From a general viewpoint, the concept of large eddy simulation (LES) seems to be without alternative [1,2,3,4,5,6]. Flow resolution provided via LES is a requirement to properly deal with many flow simulations because of our inability to accurately model such flows. Simultaneously, LES is often found to be computationally more efficient than direct numerical simulation (DNS). But despite the major contributions of LES to turbulent flow analyses and predictions, LES still faces significant challenges. Compared to Reynolds-averaged Navier–Stokes (RANS) methods which include transport equations for the typical scale of turbulent motions, an essential characteristics of classical LES is to provide such required scale information algebraically by using the filter width Δ as model length scale. The latter concept is known to fail outside of the inertial range (close to DNS or RANS regimes) [1,7,8]. From a practical viewpoint, there are also significant questions about LES. In regard to complex high Reynolds number (Re) flow simulations, LES is usually extremely expensive computationally [9,10,11], and these costs drastically increase with Re [12]. Arguably, one of the biggest concerns about LES is the missing involvement of a reliable measure of its resolution ability. As is well known, the assessment of the actual LES resolution represents a rather difficult question [13,14].

The hybridization of LES with computationally much more efficient RANS methods is seen to be the most promising way to overcome the problems described in the preceding paragraph. A variety of ways was suggested to accomplish such a hybridization. The first way is wall-modeled LES (WMLES) [2,5,6,15,16,17,18,19,20,21,22,23,24,25,26,27], where RANS components are involved close to solid walls. Variations of this approach given by the development of Reynolds-stress-constrained LES (RSC-LES) [28,29,30,31,32,33,34,35,36,37,38,39] are discussed elsewhere [40]. The second way is given by detached eddy simulation (DES) [41,42,43,44,45,46,47,48,49,50,51,52], where the performance of RANS models is improved by switching from the RANS turbulence length scale applied close to the wall to a much smaller LES-type length scale away from the wall. Many alternative methods have been suggested [40,53], including unified RANS-LES (UNI-LES) [54,55,56,57,58,59,60], partially averaged Navier–Stokes (PANS) [61,62,63,64,65,66,67,68,69,70,71,72], partially integrated transport modeling (PITM) [73,74,75,76,77,78,79,80,81,82,83], and scale adaptive simulation (SAS) methods [52,84,85,86,87,88,89]. Although these hybrid RANS-LES methods can reduce the computational cost of LES, their use also brings up significant questions. Typical problems are given by the variety of available model and simulation settings (possibly leading to a significant uncertainty of predictions), or the discrepancy between prescribed and actual flow resolution (possibly leading to significant performance shortcomings). Such issues apply to aerospace and wind energy problems but also to a variety of other problems, for example, mesoscale and microscale modeling in regard to atmospheric simulations and many technical applications [90,91,92]. Also, the use of machine learning (ML) methods has become increasingly popular. Such developments are clearly promising, but there is currently no indication that the use of ML methods in regard to the problem considered relates to essential methodological improvements [93,94,95,96,97,98,99,100,101,102,103,104,105,106,107,108,109,110,111,112,113,114].

The intention of this paper is to use exact mathematical analysis to identify reasons for the shortcomings of LES and hybrid RANS-LES and to present alternative methods, referred to as continuous eddy simulation (CES), which are not affected by such issues. There are several differences to previous related work [9,10,11,40,115,116,117,118,119,120,121,122]. First, a novel interpretation of the analytical approach is presented as a variant of minimizing the uncertainty (measured by the entropy), in line with the corresponding use of the entropy to design statistically most likely probability density function (PDF) methods [123]. Second, previously obtained results are generalized by the separate consideration of a turbulence scale equation, which is very beneficial to identify the general hybridization mechanism mathematically obtained in this way. Third, a relevant practical problem is addressed: the hybridization under conditions where a transport equation for turbulence scale variables is unavailable [there are several usually applied codes that do not involve dissipation transport equations, as is currently the case with the widely used Weather Research and Forecasting Model (WRF) dealing with atmospheric flow simulations]. Fourth, basic disadvantages of existing usually applied computational methods and advantages of novel minimal error simulation methods are identified. The paper is organized in the following way. Exact analysis results are presented in Section 2. Implications for computational methods are presented in Section 3. Evidence for the benefits of mathematically based minimal error simulation methods is presented in Section 4, followed by the conclusions in Section 5.

## 2. Analysis Results: Minimal Error Simulation Methods

### 2.1. Theoretical Basis

The analysis presented next does not present a model but a model design methodology that can be applied to many turbulence model structures. More specifically, analysis as presented here cannot be applied to any equation structure, e.g., to determine source terms in the momentum equation. This analysis applies to equation structures that establish relationships between model variables (like the modeled kinetic energy *k*) and model parameters (like $\psi_{\alpha }$, see Equation (2)). It is worth noting, however, that this approach covers the usually applied basis for both LES and hybrid RANS-LES methods.

We consider the incompressible continuity equation $𝜕{\tilde{U}}_{i}/𝜕x_{i}=0$ and momentum equation

(1) $$\frac{D{\tilde{U}}_{i}}{Dt}=-\frac{𝜕(\tilde{p}/\rho +2k/3)}{𝜕x_{i}}+2\frac{𝜕\left(\nu +\nu_{t}\right){\tilde{S}}_{ik}}{𝜕x_{k}}.$$

Here, $D/Dt=𝜕/𝜕t+{\tilde{U}}_{k}𝜕/𝜕x_{k}$ denotes the filtered Lagrangian time derivative, and the sum convention is used throughout this paper. ${\tilde{U}}_{i}$ refers to the *i*th component of the spatially filtered velocity. We have here the filtered pressure $\tilde{p}$, ρ is the constant mass density, *k* is the modeled energy, ν is the constant kinematic viscosity, and ${\tilde{S}}_{ij}=\left(𝜕{\tilde{U}}_{i}/𝜕x_{j}+𝜕{\tilde{U}}_{j}/𝜕x_{i}\right)/2$ is the resolved rate-of-strain tensor. The modeled viscosity is given by $\nu_{t}=C_{\mu }k\tau =C_{\mu }k^{2}/\epsilon$. Here, ϵ is the modeled dissipation rate of modeled energy *k*, τ=k/ϵ is the dissipation time scale, and $C_{\mu }$ has a standard value $C_{\mu }=0.09$. For *k*, we consider the transport equation

(2) $$\frac{Dk}{Dt}=P-\psi_{\alpha }\epsilon +D_{k}.$$

The diffusion term reads $D_{k}=𝜕\left[\nu_{t}\;𝜕k/𝜕x_{j}\right]/𝜕x_{j}$, and $P=\nu_{t}S^{2}$ is the production of *k*, where $S={\left(2{\tilde{S}}_{mn}{\tilde{S}}_{nm}\right)}^{1/2}$ is the characteristic shear rate. The variable $\psi_{\alpha }$ appears here in contrast to usually applied RANS equations where $\psi_{\alpha }=1$ is applied.

Although different, the type of analysis presented here shows common features with the derivation of an optimal PDF based on maximizing the related entropy, which is also accomplished via variational analysis. In the following, hybridization errors λ given by residuals of Equation (2),

(3) $$\lambda =\frac{Dk}{Dt}-P+\psi_{\alpha }\epsilon -D_{k},$$

play the central role of the analysis applied. In particular, normalized hybridization errors $\lambda^{*}$ are applied (as given by $\epsilon \lambda /k^{3}$ in Equation (5)). Although not applied below to simplify the presentation, such normalized errors can be always made nondimensional by applying appropriate total variables, which are unaffected by variations. The normalized errors $\lambda^{*}$ are measures of uncertainty (of the hybridization uncertainty) in the same way as the entropy *E* is introduced as measure of uncertainty. Formally, we may state, therefore,

(4) $$E=\lambda^{*}.$$

The requirement for vanishing first order variations of these normalized errors then identifies extremal relations. The methods obtained in this way represent minimal error simulation methods.

A core component of the following discussions is the understanding of (the interaction of) resolved and modeled motions [40]. Here, the term modeled motions relates to model variables which are determined via the turbulence model applied, as given by Equation (2). Using appropriate computational grids, such simulation methods can produce fluctuations (of velocities, kinetic energy and other variables), which represent resolved motion (which is not explicitly modeled but produced by the model). Such resolved motion can be statistically processed and measured by averaging simulation results. The involvement of resolved motion plays an essential role in simulations: it enables proper simulations of separated turbulent flows, which appear in most practical applications. The basic goal of the methods presented here is the proper involvement of both modeled and resolved motion. In particular, the goal is to set up an appropriate interaction of both types of motion. The latter is a requirement to correctly manage transitions between almost modeled (RANS-type) and almost resolved (LES-type) flow regimes: the model contribution needs to decrease (increase) if there is a lot of (little) resolved motion. Technically, the latter can be accomplished via variations of $\psi_{\alpha }$. Different ways to deal with this question (different hybridizations which are summarized in Table 1) are described in the following subsections and in Appendix A, which presents a relevant modification.

Correspondingly, there are two types of variables: model variables and total variables, referred to by the subscript tot. Total variables involve both model and resolved contributions [122]. The variables considered here are the modeled kinetic energy *k*, dissipation rate ϵ, time scale τ=k/ϵ, and length scale $L=k^{3/2}/\epsilon =k^{1/2}\tau$. Fraction $L_{+}=L/L_{tot}$ specifies the amount of resolved motion in regard to the variable considered; the same applies to $k_{+}=k/k_{tot}$ and $\epsilon_{+}=\epsilon /\epsilon_{tot}$. The latter fractions are bounded by zero and one, e.g., $0\leq L_{+}\leq 1$. Here, $L_{+}$, $k_{+}$, $\tau_{+}$ values of zero refer to fully resolved flow, and $L_{+}$, $k_{+}$, $\tau_{+}$ values of unity refer to fully modeled flow. A relevant technical detail is the calculation of $L_{+}$ in simulations ($k_{+}=k/k_{tot}$ and $\tau_{+}=\tau /\tau_{tot}$ are calculated correspondingly). The modeled contribution is calculated by $L={\langle k\rangle }^{3/2}/\langle \epsilon \rangle$, where the brackets refer to averaging in time. The total length scale is calculated correspondingly by $L_{tot}=k_{tot}^{3/2}/\epsilon_{tot}$. Corresponding to $k_{tot}=\langle k\rangle +k_{res}$, $\epsilon_{tot}$ is the sum of modeled and resolved contributions, $\epsilon_{tot}=\langle \epsilon \rangle +\epsilon_{res}$. Here, the resolved contributions are calculated by $k_{res}=\left(\langle {\tilde{U}}_{i}{\tilde{U}}_{i}\rangle -\langle {\tilde{U}}_{i}\rangle \langle {\tilde{U}}_{i}\rangle \right)/2$, $\epsilon_{res}=\nu \left(\langle 𝜕{\tilde{U}}_{i}/𝜕x_{j}𝜕{\tilde{U}}_{i}/𝜕x_{j}\rangle -\langle 𝜕{\tilde{U}}_{i}/𝜕x_{j}\rangle \langle 𝜕{\tilde{U}}_{i}/𝜕x_{j}\rangle \right)$.

To obtain methods that can function well in applications, the hybridization parameter $\psi_{\alpha }$ as obtained via analysis of Equation (3) should be a local function of turbulence variables themselves. Thus, $\psi_{\alpha }$ should not depend on Dk/Dt and the turbulent transport term $D_{k}$. Technically, the influence of these terms can be excluded by applying variational analysis to normalized errors (see below). Variations are denoted by δ. Dk/Dt is characterized by δ(Dk/Dt)=[δk/k]Dk/Dt, and $D_{k}$ is characterized by $\delta D_{k}=\left[3\delta k/k-\delta \epsilon /\epsilon \right]D_{k}$, which means there is no normalization that enables the simultaneous disappearance of Dk/Dt and $D_{k}$.

- One way to deal with this (which corresponds to PANS and PITM) is to neglect $D_{k}$ in regard to the $\psi_{\alpha }$ calculation, leading to the significant shortcoming that this hybridization approach can only be applied to homogeneous flows. Thus, this approach is not considered here. It is worth noting, however, that this approach results in the same results as reported in Section 2.2 because the corresponding variations of $D_{k}$ (and $D_{\epsilon }$ if an ϵ-equation is involved) disappear.
- Then, there are two possibilities: analysis including Dk/Dt in conjunction with a redefinition of $D_{k}$, or the inclusion of $D_{k}$ in conjunction with the neglect of Dk/Dt. These two options (leading to O1 and O2 results presented in Table 1) are considered in Section 2.2 and Section 2.3.
- A third option (a specification of option O2 leading to O3 results presented in Table 1) is considered in the Appendix A.

It is worth noting that the following analysis involves a relevant assumption made throughout this paper: we assume that the energy partition (δk/k and δϵ/ϵ) does not change in space and time. This assumption is not a restriction but a desired stability requirement. It ensures that physically equivalent flow regions are equally resolved without significant oscillations of δk/k and δϵ/ϵ [9,121,122].

### 2.2. Exact Hybridization

According to the choices described in the preceding paragraph, we consider a redefinition of $D_{k}$ in Equation (2) given by $D_{k}^{*}=𝜕\left[\nu_{t,tot}\;𝜕k/𝜕x_{j}\right]/𝜕x_{j}$, i.e., the replacement of $\nu_{t}$ in $D_{k}$ by $\nu_{t,tot}$. The corresponding analysis leads to the O1 formulas in Table 1.

First, we consider the case of making no assumption on ϵ. The appropriately normalized error of this modified Equation (2) reads, then,

(5) $$\frac{\lambda }{k}=\frac{1}{k}\left[\frac{Dk}{Dt}-P+\epsilon \psi_{\alpha }-D_{k}^{*}\right],$$

where the variations related to Dk/Dt and $D_{k}$ disappear:

(6) $$\delta \left[\frac{1}{k}\frac{Dk}{Dt}\right]=\frac{1}{k}\frac{Dk}{Dt}\left[\frac{\delta (Dk/Dt)}{Dk/Dt}-\frac{\delta k}{k}\right]=0,\;\delta \left[\frac{D_{k}^{*}}{k}\right]=\frac{D_{k}^{*}}{k}\left[\frac{\delta D_{k}^{*}}{D_{k}^{*}}-\frac{\delta k}{k}\right]=0.$$

By using ϵ=k/τ, the requirement of a vanishing first-order variation of the normalized error implies, then,

(7) $$\delta \left[\frac{\psi_{\alpha }}{\tau }\right]=\delta \left[\frac{P}{\epsilon \tau }\right].$$

By involving $\tau_{+}=k_{+}/\epsilon_{+}$, the integration of this equation from the RANS state (where $\psi_{\alpha }=1$, the variables involved are total variables indicated by the subscript tot) to a state with a certain level of resolved motion provides

(8) $$\frac{\psi_{\alpha }}{\tau }-\frac{1}{\tau_{tot}}=\frac{P}{\epsilon \tau }-\frac{P_{tot}}{\epsilon_{tot}\tau_{tot}},\;\mathrm{or}\;\psi_{\alpha }=\frac{P}{\epsilon }-\tau_{+}\left(\frac{P_{tot}}{\epsilon_{tot}}-1\right).$$

The latter relation can be specified by involving an ϵ-equation, which can be used to rewrite the production *P*,

(9) $$\frac{D\epsilon }{Dt}=C_{\epsilon_{1}}\frac{\epsilon^{2}}{k}\left(\frac{P}{\epsilon }-\alpha \right)+D_{\epsilon }^{*},\;\mathrm{or}\;\frac{P}{k}=\alpha \frac{\epsilon }{k}+\frac{1}{C_{\epsilon_{1}}\epsilon }\left[\frac{D\epsilon }{Dt}-D_{\epsilon }^{*}\right].$$

To be consistent with $D_{k}$, $D_{\epsilon }^{*}$ also involves the total viscosity, $D_{\epsilon }^{*}=𝜕\left[\left(\nu_{t,tot}/\sigma_{\epsilon }\right)\;𝜕\epsilon /𝜕x_{j}\right]/𝜕x_{j}$. The variation of the last term disappears for the case considered:

(10) $$\delta \left[\frac{D\epsilon /Dt}{C_{\epsilon_{1}}\epsilon }\right]=\frac{D\epsilon /Dt}{C_{\epsilon_{1}}\epsilon }\left[\frac{\delta (D\epsilon /Dt)}{D\epsilon /Dt}-\frac{\delta \epsilon }{\epsilon }\right]=0,\;\delta \left[\frac{D_{\epsilon }^{*}}{C_{\epsilon_{1}}\epsilon }\right]=\frac{D_{\epsilon }^{*}}{C_{\epsilon_{1}}\epsilon }\left[\frac{\delta D_{\epsilon }^{*}}{D_{\epsilon }^{*}}-\frac{\delta \epsilon }{\epsilon }\right]=0.$$

Instead of Equation (7), we obtain

(11) $$\delta \left[\frac{\psi_{\alpha }}{\tau }\right]=\delta \left[\frac{\alpha }{\tau }\right].$$

Similar to Equation (8), the integration of this equation leads to

(12) $$\frac{\psi_{\alpha }}{\tau }-\frac{1}{\tau_{tot}}=\frac{\alpha }{\tau }-\frac{\alpha }{\tau_{tot}},\;\mathrm{or}\;\psi_{\alpha }=\alpha -\tau_{+}\left(\alpha -1\right).$$

The hybridization mechanism presented in this way can be seen very well by combining $Dk/Dt=P-\psi_{\alpha }\epsilon +D_{k}^{*}$ and $\psi_{\alpha }=P/\epsilon -\tau_{+}\left(P_{tot}/\epsilon_{tot}-1\right)$,

(13) $$\frac{Dk}{Dt}=\tau_{+}\frac{\epsilon }{\epsilon_{tot}}\left(P_{tot}-\epsilon_{tot}\right)+D_{k}^{*},\;\mathrm{or},\;\frac{Dk}{Dt}=k_{+}\left(P_{tot}-\epsilon_{tot}\right)+D_{k}^{*},$$

where $\tau_{+}\epsilon_{+}=k_{+}$ is applied. This reveals the exact mathematically determined hybridization mechanism: an increased amount of resolved motion decreases both production and dissipation until *k* almost vanishes, leading to a vanishing modeled viscosity $\nu_{t}$ (corresponding to the DNS limit). The inclusion of the ϵ Equation (9) leads to similar features. The use of $\psi_{\alpha }=\alpha -\tau_{+}\left(\alpha -1\right)$ in $Dk/Dt=P-\psi_{\alpha }\epsilon +D_{k}^{*}$ implies

(14) $$\frac{Dk}{Dt}=P-\alpha \epsilon +\tau_{+}\epsilon_{+}\left(\alpha -1\right)\epsilon_{tot}+D_{k}^{*},\;\mathrm{or},\;\frac{Dk}{Dt}=P-\alpha \epsilon +k_{+}\left(\alpha \epsilon_{tot}-\epsilon_{tot}\right)+D_{k}^{*}.$$

The correspondence between Equations (13) and (14) can be seen by accounting for the fact that P∝αϵ according to Equation (9) in regard to production and dissipation terms. It is worth noting that Equation (14) is the exact consequence of involving Equation (9); no approximations are applied.

### 2.3. Reduced Exact Hybridization

The methods reported in the preceding subsection (in O1 in Table 1) are theoretically fully convincing; the disadvantage is the involvement of total variables like the total modeled viscosity $\nu_{t,tot}$ in turbulent transport terms $D_{k}^{*}$ (and possibly $D_{\epsilon }^{*}$). The calculation of such terms is not trivial; these quantities may have a large range of variations. Therefore, from a computational viewpoint, neglecting substantial derivatives (only in regard to the calculation of hybridization parameters) is highly beneficial. The latter assumption (leading to bounded variations of resolution measures like $0\leq L_{+}^{2}\leq 1$) was proven to be fully justified in all applications considered so far.

Correspondingly, we consider the normalized hybridization error λ without involving Dk/Dt in Equation (3), without making any assumptions on ϵ,

(15) $$\frac{\epsilon \lambda }{k^{3}}=-\frac{\epsilon P}{k^{3}}+\frac{\epsilon^{2}\psi_{\alpha }}{k^{3}}-\frac{\epsilon D_{k}}{k^{3}}.$$

The normalization ($\epsilon /k^{3}$) is motivated by the fact that the variation of the last term vanishes,

(16) $$\delta \left[\frac{\epsilon D_{k}}{k^{3}}\right]=\frac{\epsilon D_{k}}{k^{3}}\left(\frac{\delta \epsilon }{\epsilon }-3\frac{\delta k}{k}+\frac{\delta D_{k}}{D_{k}}\right)=0,$$

because the variation of $D_{k}$ implies a zero parenthesis term. We require a zero first-order variation of $\epsilon \lambda /k^{3}$, which leads to

(17) $$\delta \left[\frac{\epsilon^{2}\psi_{\alpha }}{k^{3}}\right]=\delta \left[\frac{\epsilon P}{k^{3}}\right],\;\mathrm{or}\;\delta \left[\frac{\psi_{\alpha }}{L^{2}}\right]=\delta \left[\frac{P}{\epsilon L^{2}}\right].$$

Here, Equation (16) is taken into account, and the last expression involves the characteristic turbulence length scale $L=k^{3/2}/\epsilon$. The latter equation can be integrated from the RANS state (where $\psi_{\alpha }=1$) to a state with a certain level of resolved motion,

(18) $$\frac{\psi_{\alpha }}{L^{2}}-\frac{1}{L_{tot}^{2}}=\frac{P}{\epsilon L^{2}}-\frac{P_{tot}}{\epsilon_{tot}L_{tot}^{2}},\;\mathrm{or}\;\psi_{\alpha }=\frac{P}{\epsilon }-L_{+}^{2}\left(\frac{P_{tot}}{\epsilon_{tot}}-1\right).$$

It is worth noting that $P_{tot}=\nu_{t,tot}S^{2}$, where *S* refers to the resolved shear rate.

A modification of this calculation arises if the usual transport equation for ϵ is involved:

(19a) $$\begin{array}{c} \frac{D\epsilon }{Dt}=C_{\epsilon_{1}}\frac{\epsilon^{2}}{k}\left(\frac{P}{\epsilon }-\alpha \right)+D_{\epsilon },\;\mathrm{where}\; \end{array}$$

(19b) $$\begin{array}{c} \delta \left[\frac{D_{\epsilon }}{C_{\epsilon_{1}}k^{2}}\right]=\frac{D_{\epsilon }}{C_{\epsilon_{1}}k^{2}}\left(\frac{\delta D_{\epsilon }}{D_{k}}-2\frac{\delta k}{k}\right)=0. \end{array}$$

The diffusion term is given by $D_{\epsilon }=𝜕\left[\left(\nu_{t}/\sigma_{\epsilon }\right)\;𝜕\epsilon /𝜕x_{j}\right]/𝜕x_{j}$: the relevant variation of $D_{\epsilon }$ arising from the structure of $D_{\epsilon }$ is involved in Equation (19b). Here, $C_{\epsilon_{1}}$, $C_{\epsilon_{2}}$, $\alpha =C_{\epsilon_{2}}/C_{\epsilon_{1}}$ and $\sigma_{\epsilon }$ are model parameters: usually applied values are $\sigma_{\epsilon }=1.3$, $C_{\epsilon_{1}}=1.44$, $C_{\epsilon_{2}}=1.92$, resulting in α=1.33. By neglecting the substantial derivative Dϵ/Dt in correspondence with neglecting Dk/Dt above (only in regard to the calculation of the hybridization parameter $\psi_{\alpha }$), this ϵ equation provides a relation for the production *P* given by

(20) $$\frac{\epsilon P}{k^{3}}=\alpha \frac{\epsilon^{2}}{k^{3}}-\frac{D_{\epsilon }}{C_{\epsilon_{1}}k^{2}},\;\mathrm{or},\;\frac{P}{\epsilon L^{2}}=\frac{\alpha }{L^{2}}-\frac{D_{\epsilon }}{C_{\epsilon_{1}}k^{2}}.$$

The variation of the last term vanishes; see Equation (19b). The replacement of $P/[\epsilon L^{2}]$ according to Equation (20) in Equation (17) then implies

(21) $$\delta \left[\frac{\psi_{\alpha }}{L^{2}}\right]=\alpha \delta \left[\frac{1}{L^{2}}\right].$$

As above, we integrate this equation from the RANS state ($\psi_{\alpha }=1$) to a state with a certain level of resolved motion,

(22) $$\frac{\psi_{\alpha }}{L^{2}}-\frac{1}{L_{tot}^{2}}=\frac{\alpha }{L^{2}}-\frac{\alpha }{L_{tot}^{2}},\;\mathrm{or}\;\psi_{\alpha }=\alpha -L_{+}^{2}\left(\alpha -1\right).$$

Similar to Equations (13) and (14), we look at the implied hybridization mechanism without/with involving the ϵ-equation, respectively. Equation $Dk/Dt=P-\psi_{\alpha }\epsilon +D_{k}$ combined with $\psi_{\alpha }=P/\epsilon -L_{+}^{2}\left(P_{tot}/\epsilon_{tot}-1\right)$ leads to

(23) $$\frac{Dk}{Dt}=P-\left[P/\epsilon -L_{+}^{2}\left(P_{tot}/\epsilon_{tot}-1\right)\right]\epsilon +D_{k},\;\mathrm{or},\;\frac{Dk}{Dt}=L_{+}^{2}\epsilon_{+}\left(P_{tot}-\epsilon_{tot}\right)+D_{k}.$$

Otherwise, equation $Dk/Dt=P-\psi_{\alpha }\epsilon +D_{k}$ combined with $\psi_{\alpha }=\alpha -L_{+}^{2}\left(\alpha -1\right)$ leads to

(24) $$\frac{Dk}{Dt}=P-\left[\alpha -L_{+}^{2}\left(\alpha -1\right)\right]\epsilon +D_{k},\;\mathrm{or},\;\frac{Dk}{Dt}=P-\alpha \epsilon +L_{+}^{2}\epsilon_{+}\left(\alpha \epsilon_{tot}-\epsilon_{tot}\right)+D_{k}.$$

Compared to the corresponding O1 formulas, we observe the same hybridization mechanism where $\tau_{+}$ is replaced by $L_{+}^{2}$, which is implied by exact analysis.

## 3. Implications for Computational Methods

### 3.1. Implications for LES

LES aims at resolving most of the turbulent flow using a relatively small turbulent viscosity $\nu_{t}=C_{\mu }k^{1/2}L$ in conjunction with a relatively fine grid. The relationship to the minimal error methods presented here can be seen by a comparison of corresponding dissipation rates. We use $Dk/Dt=P-\psi_{\alpha }\epsilon +D_{k}$ for that, which involves the same diffusion coefficient. The dissipation rate applied in standard LES is $\epsilon =k^{3/2}/\Delta$, with Δ being the filter width. The dissipation rate in $Dk/Dt=P-\psi_{\alpha }\epsilon +D_{k}$ is $\psi_{\alpha }\epsilon =\psi_{\alpha }k^{3/2}/L$, where $\psi_{\alpha }=\alpha -L_{+}^{2}\left(\alpha -1\right)$. The equality of both dissipation rates reveals the applicability conditions for the LES expression: it requires

(25) $$\frac{1}{\Delta }=\frac{1}{L}\left[\alpha -L_{+}^{2}\left(\alpha -1\right)\right],\;\mathrm{or}\;L_{+}=\Delta_{+}\left[\alpha -L_{+}^{2}\left(\alpha -1\right)\right].$$

The last expression results from multiplication with $L_{tot}$, and we introduce $\Delta_{+}=\Delta /L_{tot}$. This equation represents a quadratic equation in $L_{+}$ which is solved by

(26) $$L_{+}=\sqrt{\frac{\alpha }{\alpha -1}+\frac{1}{4{\left(\alpha -1\right)}^{2}\Delta_{+}^{2}}}-\frac{1}{2\left(\alpha -1\right)\Delta_{+}}\;\underset{\Delta_{+}<<1}{\to }\;L_{+}=\alpha \Delta_{+},$$

where $0\leq \Delta_{+}\leq 1$ is considered, which ensures $0\leq L_{+}\leq 1$. For small $\Delta_{+}$, Equation (26) is reduced to $L_{+}=\alpha \Delta_{+}$ (or L=αΔ), as may be seen by neglecting the $L_{+}^{2}$ term in Equation (25). Thus, compared to the minimal error concept, the LES concept is to replace the scale calculation by setting L=αΔ, where Δ is sufficiently small, which leads to a small $\nu_{t}=C_{\mu }k^{1/2}L$. The classical LES concept to assume L=αΔ is known to fail outside of the inertial range (close to DNS or RANS regimes) [1,7,8]. Explicit evidence for that can be found elsewhere [7,8]; see, in particular, the discussion in Ref. [7] related to Figure 11 there. More specifically, we observe the following:

C1. **Improper resolution variation:** The classical LES concept to set L=αΔ enables flow resolution via grid refinements. However, a grid refinement (smaller Δ) has opposite effects on production and dissipation in the *k*-equation because of $P=C_{\mu }k^{1/2}LS^{2}$ and $\epsilon =k^{3/2}/L$: the smaller L=αΔ decreases the production and increases the dissipation of *k*, leading to a drastic reduction in *k*. This is in contradiction to Equation (23) and the other equations reported in Section 2. The exact derivation of these equations reveals the simultaneous damping of both production and dissipation if the amount of resolved motion increases. The same mechanism creates the well-known failure of LES on relatively coarse grids (as always given for very high Re flows): the relatively large Δ implies an overestimation of production and underestimation of dissipation in the *k*-equation.
C2. **Huge cost:** One consequence of the LES resolution concept is the huge cost of LES to ensure appropriate flow resolution. Another consequence is that LES does not involve an explicit measure of the flow resolution, which would provide guidance about the actual flow resolution. This implies the difficult question of how to evaluate the LES flow resolution [13,14].
C3. **Alternative:** These problems can be avoided by replacing the filter width by the turbulence length scale *L* calculated by involving a scale equation in accordance with minimal error equations. In particular, in contrast to the usual LES concept, *L* can physically correctly represent the size of turbulence structures. The LES resolution is explicitly specified via the known $L_{+}$.

### 3.2. Implications for WMLES and DES Type Models

Hybrid RANS-LES methods, first of all DES and WMLES methods, were developed to overcome the (near-wall resolution) issues of LES. The functioning of these methods is very different from the methods described here; see O1. The predominant strategy is the design of equations that involve both LES and RANS components. This takes place, for example, by the inclusion of relatively small LES length scales (modeled viscosities) in RANS which become active away from walls, or the switch from LES to RANS turbulent viscosities near walls. Because of their design, such viscosity-switching methods are known to suffer from functionality issues: results depend on the use of different (equilibrium or non-equilibrium) wall models, definitions of regions where different models and grids are applied, different mesh distributions, and set-up options to manage the information exchange between such different flow regions. These equations do not involve resolution indicators. We observe the following:

C4. **Improper resolution variation:** WMLES and DES methods are subject to the LES resolution issues reported above because of their explicit inclusion of LES-type equations. The basic goal is to extend the applicability of LES to coarse-grid simulations. But on coarse grids, the LES scaling with Δ represents an unphysical concept [1,7,8], and the resolution mechanism functioning becomes increasingly incorrect (see C1).
C5. **Imbalanced resolution transition:** Even more importantly, such methods are known to often inadequately handle transitions from modeled to resolved motion and vice versa. The latter is also a consequence of their design: the switch of modeled viscosities without accounting for the actual amount of flow resolution is an insufficient concept. The latter requires empirical matching methods to adjust to different flows [10,11].
C6. **Alternative:** In contrast to the functionality issues of usually applied RANS-LES, in particular DES and WMLES, the methods reported in O2 in Table 1 are not affected by corresponding problems. The underlying RANS model is modified by the mathematical hybridization approach. There are no further model set-up options in regard to setting up the hybridization or in regard to dealing with performance issues related to transitions between modeled and resolved motions.

### 3.3. Implications for PANS and PITM-Type Models

Another strategy to deal with the LES near-wall resolution problems is to partially follow the O1, O2 results. There exist methods which show some technical similarities to the methods reported here; see Refs. [7,8,124]. The basic idea is the damping of modeled viscosity via $\nu_{t}=k_{+}\nu_{t,tot}$. This assumption is not too far from the implications of methods reported here: by taking reference to Equation (13), we have $k_{+}P_{tot}=k_{+}\nu_{t,tot}S^{2}$. However, in comparison to the exact results reported here, this empirical approach misses the corresponding damping of dissipation; see Equation (13). This may imply physically incorrect variations of *k* and ϵ. Alternatives were presented in terms of PANS and PITM methods. Instead of $\psi_{\alpha }=\alpha -L_{+}^{2}\left(\alpha -1\right)$ (see O2 in Table 1), these methods are based on $\psi_{\alpha }=\alpha -R\left(\alpha -1\right)$, where $R=k_{+}$ (see the last paragraph of Section 2.1, first bullet point). More specifically, $\epsilon_{+}=1$ is assumed, and $R=k_{+}$ is approximated: *R* is a prescribed constant (PANS) or $R=1.06\Delta_{+}^{2/3}$ (PITM). We observe the following:

C7. **Improper resolution variation:** The consideration of $R=k_{+}$ has an essential disadvantage: this hybridization is only applicable to homogeneous flows (see Section 2.1). This approach is in contradiction with the goal of hybrid RANS-LES to provide better predictions of nonhomogeneous flows.
C8. **Resolution mismatch:** The significant difference to the minimal error methods reported here is the functioning of methods. In PANS and PITM methods, there is a certain desired amount of resolved motion imposed on the simulation by the model set-up (the *R* applied). Because of the approximation of $k_{+}$ applied, there is no feedback between resolved and modeled motion, and the imposed resolution is often not realized computationally. Similar to corresponding problems of WMLES and DES, this discrepancy between modeled and resolved motion may imply significant model performance issues; see detailed analyses reported elsewhere [9,10,11].
C9. **Alternative:** In correspondence to C6 presented in regard to WMLES and DES methods, these problems are overcome by the methods presented here. In particular, the minimal error concept ensures a correct hybridization mechanism. Because of the active interaction of resolved and modeled motion, corresponding imbalances seen in PANS and PITM methods are avoided.

## 4. Simulation Results

Further evidence for the facts reported in Section 3 (the simulation performance problems C2, C5, C8 of existing simulation methods and advantages of CES methods) is provided next via results obtained by simulations of three complex high Re turbulent flows. The flows considered, issues of existing simulation methods, and benefits of CES methods are addressed in the next three subsections. Corresponding CES analyses involve the consideration of different CES versions [9,10,11]. The following results are reported in regard to the CES-KOS (or simply KOS) CES version. Here, KO refers to the use of a k−ω model, and S refers to the hybridization in the scale equation. A discussion of the equivalence of different CES hybridizations can be found elsewhere [9].

### 4.1. Flows Considered

**Figure 1:**
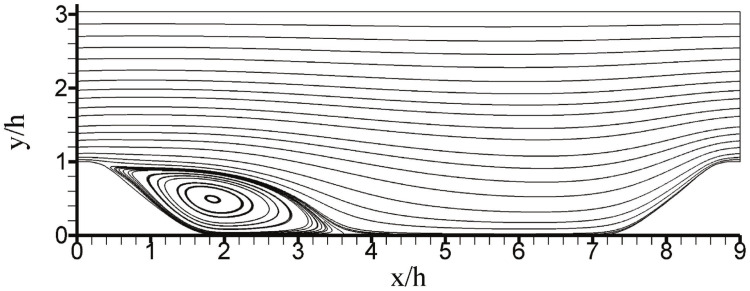
Velocity streamlines seen in periodic hill flows: results obtained by continuous eddy simulation at Re=37,000. Reprinted with permission from Ref. [9]. Copyright 2020 AIP Publishing.

One of the applications of CES methods is the simulation of periodic hill flows as illustrated in Figure 1 [9]. This flow is a channel flow involving periodic restrictions. This flow, which is used a lot for the evaluation of turbulence models [40], involves features such as separation, recirculation, and natural reattachment [125,126]. The size of the computational domain is $L_{x}=9h$, $L_{y}=3.035h$, and $L_{z}=4.5h$ in the streamwise *x*, wall normal *y*, and spanwise *z* directions, respectively: *h* refers to the hill height. At the bottom and top, the channel is constrained by solid walls. No-slip and impermeability boundary conditions are used at these walls. Periodic boundary conditions are applied in streamwise and spanwise directions. The flow simulations were performed for a wide range of Re ranging from Re=37 K up to Re=500 K using grids involving between 120 K and 500 K grid points (these grids are denoted by $G_{120}$ and $G_{500}$, respectively). A thorough evaluation of the performance of CES methods in regard to simulating periodic hill flows at the highest Re=37 K for which experimental data for model evaluation are still available can be found elsewhere [9].

**Figure 2:**
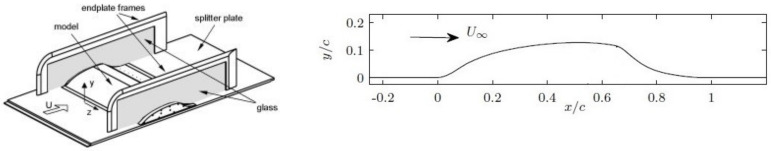
Wall-mounted hump geometry. (**left**) Experimental setup [128]; (**right**) 2D Computational layout.

Seifert and Pack developed the NASA wall-mounted hump model to investigate unsteady flow separation, reattachment, and flow control at a high Reynolds number $Re=c\rho_{ref}U_{ref}/\mu \approx 936$ K based on the chord length *c* and freestream velocity $U_{ref}$. Here, μ is the dynamic viscosity and abbreviation ref indicates the reference freestream conditions, which are determined at the axial point x/c=−2.14. The model reflects the upper surface of a 20-thick Glauert–Goldschmied airfoil that was originally designed for flow control purposes in the early twentieth century. We see in Figure 2 a strongly convex region just before the trailing edge, which induces flow separation. As a benchmark for comparison, we used the experiment conducted by Greenblatt et al. [127] without flow control [11]. This case was extensively documented on the NASA Langley Research Center’s Turbulence Modeling Resource webpage and has been widely used for evaluating different turbulence modeling techniques, as discussed in the 2004 CFD Validation Workshop.

**Figure 3:**
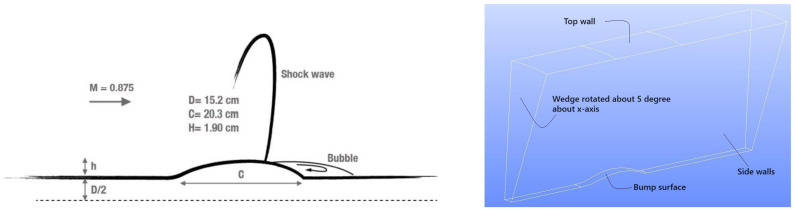
Axisymmetric transonic bump geometry: experimental and computational configuration [131,132,133].

Figure 3 shows a schematic diagram of the experimental configuration and the computational domain for the axisymmetric transonic bump considered [129,130] along with the applied boundary conditions. In particular, the Bachalo–Johnson [129] experiment provided detailed data on mean velocity profiles, the Reynolds shear stress, and surface pressure, but measurements of skin friction coefficients ($C_{f}$) were omitted. The latter were provided by a recent experiment of Lynch et al. [130]. The case considered pertains to shock-triggered boundary layer separation induced by an axially symmetric bump mounted on a slim spherical cylinder, which extends 61 cm upstream. The case reflects the upper surface of a transonic wing. It is characterized by a Mach number ($M_{\infty }$) of 0.875 and a Reynolds number Re=2.763 M relative to the airfoil’s chord length *c*. A thorough evaluation of CES simulations versus a variety of other simulation methods can be found elsewhere [10].

### 4.2. Problems of Existing Methods

**Figure 4:**
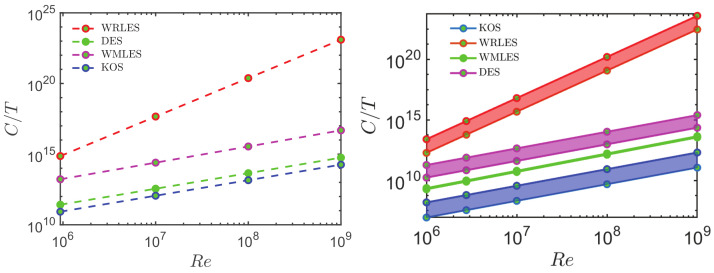
Cost scalings of CES vs. other methods [10,11]: NASA wall-mounted hump flow (**left**) and the Bachalo and Johnson axisymmetric transonic bump flow (**right**).

To illustrate the LES problem C2, we consider the computational cost of simulation methods considered. In particular, regarding the computational cost of CES methods, there are essential differences to usually applied methods [9,10,11]. The simulation costs are specified by $C=NN_{t}=TN/\Delta t$. Here, *N* is the number of grid points applied, $N_{t}$ is number of time steps performed, $T=N_{t}\Delta t$ refers to the constant total physical simulation time, and Δt is the prescribed simulation time-step. *N* and Δt are known to vary with Re according to $N=\alpha_{1}{\left(Re/Re_{0}\right)}^{\beta_{1}}$, $\Delta t=\alpha_{2}{\left(Re/Re_{0}\right)}^{-\beta_{2}}$, where $\alpha_{1}$, $\alpha_{2}$, $\beta_{1}$, and $\beta_{2}$ are constants [58,134]. Here, $Re_{0}$ is used as normalization. Implications of simulations of the NASA wall-mounted hump flow and the Bachalo and Johnson axisymmetric transonic bump flow are presented in Figure 4. As it may be seen, the simulation costs of CES are well below the cost of other methods; in particular, CES applications can be by orders of magnitude cheaper than other methods.

**Figure 5:**
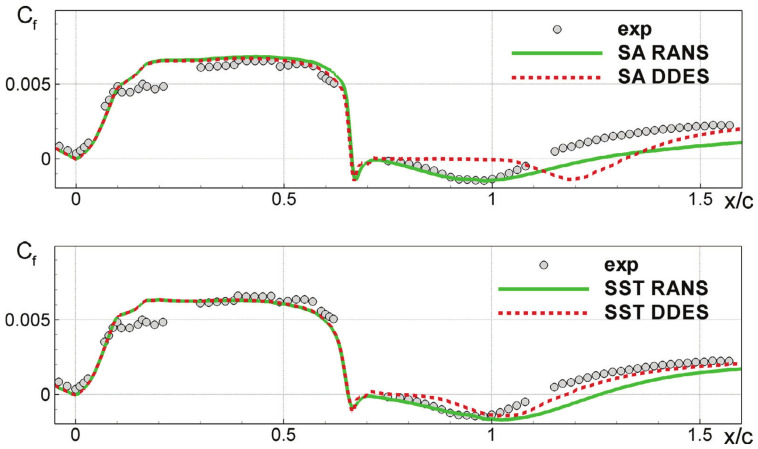
Two-dimensional NASA hump flow $C_{f}$ predictions based on several RANS and DES methods; see details in Ref. [135] [taken from Probst et al. [135] with permission].

In order to illustrate the WMLES/DES problem C5, we consider the results of applying DES methods to the NASA wall-mounted hump flow [135] as illustrated in Figure 5. In particular, these simulations involved RANS simulations with the Spalart–Allmaras (SA) turbulence model [136] and Menter’s shear stress transport (SST) model [137]. These RANS models were compared with corresponding hybrid RANS-LES models based on the delayed DES (DDES) model of Spalart et al. [47]. The unsatisfactory predictions of hybrid RANS-LES models involved (their inability to properly deal with the RANS-to-LES transition in the separated shear layer) can be clearly seen. More specifically, there are no performance improvements at all compared to the RANS predictions for x/c≤1.

**Figure 6:**
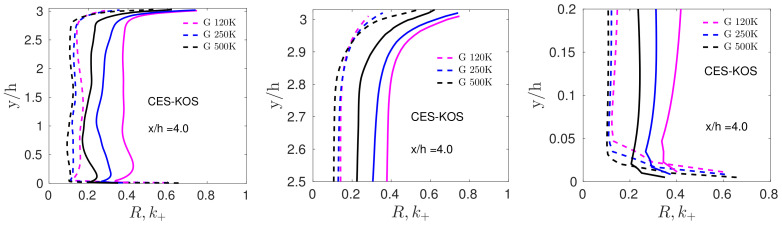
PITM concept validation for Re=37K, $G_{120},G_{250},G_{500}$ at x/h=4: full (**left**), upper wall (**middle**), and lower wall (**right**) profiles. The dashed lines show $R=C_{\Delta }\Delta_{+}^{2/3}$ and the solid lines show $k_{+}$. Reproduced from Heinz et al. [9], with the permission of AIP Publishing.

In order to illustrate the PANS/PITM problem C8, we consider corresponding results of periodic hill flow simulations presented in Ref. [9], which include a detailed comparison of CES versus PITM concepts (PANS concepts show features similar to PITM concepts). In the PITM-type model, the mode control variable $L_{+}^{2}$ was replaced by a PITM grid parameterization of $k_{+}$. Figure 6 addresses the suitability of the PITM concept subject to grid variations. Here, $k_{+}$ is the actual energy ratio seen in simulations and $R=C_{\Delta }\Delta_{+}^{2/3}$ is the prescribed energy ratio $\Delta_{+}=\Delta /L_{tot}$, Δ is the filter width ($\Delta ={\left(\Delta_{x}\Delta_{y}\Delta_{z}\right)}^{1/3}$ was applied), and $C_{\Delta }=3C_{K}/\left(2\pi^{2/3}\right)=1.06$; $C_{K}$ is the Kolmogorov constant [51]. Observations discussed in Ref. [9] are the following ones: (i) There is no indication that the prescribed *R* controls $k_{+}$, and there are significant discrepancies between prescribed and actual $k_{+}$. Relatively small variations of the prescribed *R* can imply significant $k_{+}$ variations. (ii) An unphysical behavior is found in upper and lower wall regions: a grid coarsening implies smaller $\Delta_{+}$ and actual $k_{+}$ cannot follow structural changes of imposed $k_{+}$.

### 4.3. CES Features

Essential CES features in regard to C2 (cost) and C8 (resolution mismatch) problems were already shown above: see Figure 4 and the discussion in Section 3, respectively. In particular, CES computational cost can be by orders of magnitude below the cost of existing methods, and the resolution mismatch problem simply does not exist in the frame of CES methods. Thus, we focus in the following on computational evidence of CES’s ability to properly deal with the C5 (resolution transition) problem.

**Figure 7:**
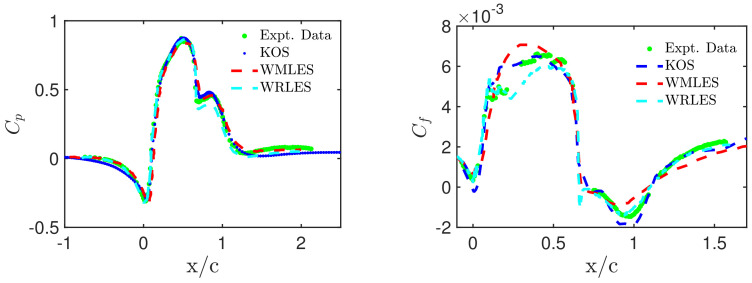
NASA wall-mounted hump flow [11]: CES-KOS, WMLES [138], and WRLES [139,140] simulation results on the $G_{4}$ grid at Re=936 K, pressure and skin-friction coefficients.

**Figure 8:**
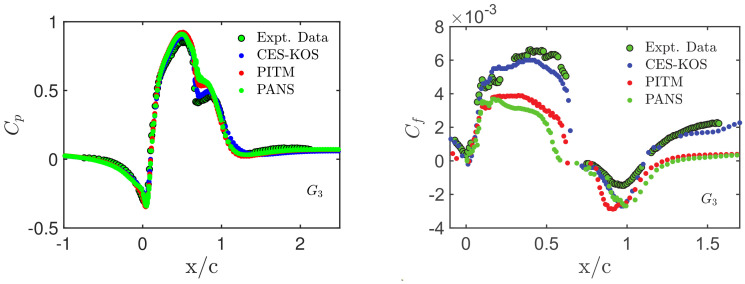
NASA wall-mounted hump flow [11]: CES-KOS, PANS, and PITM simulation results on $G_{3}$ at Re=936 K: Pressure and skin-friction coefficient profiles.

In regard to the NASA wall-mounted hump flow, a representative example of CES advantages is given in Figure 7. Pressure ($C_{p}$) and skin-friction ($C_{f}$) profiles are shown as obtained by CES (the CES-KOS version), wall-resolved LES (WRLES), and WMLES in comparison with experimental data [11]. All methods involved show a reasonable agreement with the experimental pressure coefficient profiles. The predictions from WRLES match the experimental profile downstream, and the model is capable of mimicking the dominant features of the flow. However, within the reattachment region, the second wall pressure peak is underpredicted by WRLES compared to CES-KOS and WMLES. In regard to the skin-friction coefficients, in the separation zone, from 0≤x/c≤0.65, WRLES underpredicts the skin friction coefficient, while WMLES overestimates the actual peak. In regard to post-reattachment, however, the $C_{f}$ profiles of WRLES and CES-KOS match relatively well, despite using different frameworks, mesh sizes, and grid resolutions. Overall, the CES predictions are better than the predictions of other methods, demonstrating its ability to properly transition from RANS to LES and vice versa (see the problems reported in conjunction with Figure 5). Figure 8 shows variations of pressure and skin-friction coefficients by involving PANS and PITM predictions. A detailed discussion of differences in regard to $C_{p}$ predictions can be found elsewhere [11]. The $C_{f}$ distributions reveal significant performance deficiencies of both PANS and PITM models. In particular, the comparison with Figure 5 shows that the performance of PANS and PITM methods is worse than the performance of RANS models.

**Figure 9:**
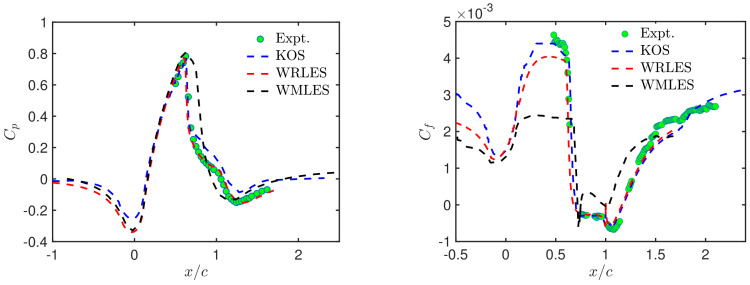
Axisymmetric transonic bump flow [10]: CES-KOS vs. WRLES [132] and WMLES [131] models, profiles of pressure and skin-friction coefficients.

Similar observations can be made in regard to the axisymmetric transonic bump flow. Figure 9 shows pressure and skin-friction distributions obtained by CES [10], WMLES [131] and WRLES [132] in comparison with experimental data [129,130]. The CES-KOS and WRLES models accurately predict pressure coefficient profiles due to their sufficient flow resolution ability. In contrast, WMLES predicts a linearly increasing pressure distribution within x/c=(0.7,1.1); it fails to accurately capture the separation zone. Both CES-KOS and WRLES show reasonable predictions of the shock location and post-shock pressure recovery. The WRLES results agree slightly better with the experimental data downstream of the bump (between x/c=1.1 and 1.3) compared to the CES-KOS model. In regard to the skin-friction coefficient distributions, WMLES significantly underestimates the skin-friction coefficient in the separation region and fails to accurately represent the post-separation flow physics. The predictions of CES-KOS and WRLES are very similar, with the exception that CES-KOS better agrees with the experimental data in the $C_{f}$ plateau region upstream of separation. Overall, CES-KOS provides the most accurate predictions, demonstrating again its ability to properly transition between RANS and LES.

## 5. Summary

The facts reported in Section 3 and Section 4 in regard to usually applied computational methods (LES, WMLES, DES, PANS, PITM) speak a clear language. There are substantial conceptual issues given by the improper resolution variations applied (see C1, C4, and C7). This implies essential practical simulation problems given by huge computational cost, imbalanced resolution transitions, and resolution mismatch (see C2, C5, and C8).

These problems were contrasted with the characteristics of exact mathematical analysis results, resulting in minimal error simulation methods. Several original research results were presented in this regard: (i) a novel interpretation of the analytical approach as a variant of minimizing the uncertainty (measured by the entropy), (ii) a general hybridization mechanism mathematically identified by separating the turbulent dissipation equation, and (iii) hybridization under conditions where a transport equation for turbulence scale variables is unavailable, which represents a relevant practical problem.

Relevant conclusions presented here can be summarized as follows.

1. It is worth noting that the conclusions obtained do not only apply to k−ϵ equation structures. Corresponding conclusions can be obtained for all usually considered turbulence models and different equation structures as given by Reynolds stress models or PDF models [117,118,119,121,122]. Again, from a methodological viewpoint, the O3 conclusions presented in Table 1 overcome relevant practical problems under conditions where the computational methodology does not include a dissipation equation. This concerns, for example, the majority of atmospheric flow simulations (we note that stratification effects can be easily included in minimal error methods [120]) and the majority of scalar transport simulations.
2. On the one hand, the separate consideration of excluding (including) an ϵ-equation as presented here is the key for the identification of the general hybridization mechanism given by the simultaneous damping of production and dissipation in the *k*-equation; see, e.g., $Dk/Dt=k_{+}\left(P_{tot}-\epsilon_{tot}\right)+D_{k}^{*}$. On the other hand, the inclusion of the ϵ-equation implies significant methodological simplifications due to P∝αϵ. This proportionality opens the way for designing correctly functioning hybrid methods which apply usually considered turbulence models modified by bounded resolution indicators: see the difference of O2 options which exclude (include) an ϵ-equation.
3. A very relevant question in regard to the following discussion is the reliability of the analysis presented. The O1 results presented in Table 1 are exact. However, this option is computationally not ideal: there are several total variables involved which are unbounded in contrast, e.g., to $L_{+}$. By including an ϵ-equation, neglecting Dk/Dt and Dϵ/Dt only in regard to the calculation of the hybridization parameter $\psi_{\alpha }$ overcomes this problem. Usually considered turbulence equations can be taken into account, where only bounded resolution indicators arise (as $0\leq L_{+}\leq 1$). Neglecting Dk/Dt and Dϵ/Dt is a usually considered, weak assumption. All applications considered so far confirm the validity of this assumptio via excellent simulation results [9,10,11]. A strong argument for the validity of this assumption is that the basic hybridization mechanism is only slightly changed in this way: $k_{+}$ is replaced by $L_{+}^{2}\epsilon_{+}$; see Table 1.
4. Arguably, the most relevant result of the analysis presented here is the identification of the mathematically implied hybridization mechanism and simultaneous identification of significant conceptual issues of usually applied computational methods in this regard (see C1, C4, and C7). The way to overcome these conceptual problems is the use of minimal error methods as presented here. The use of these methods also allows to overcome corresponding practical simulation problems (see C2, C5, and C8) given by huge computational cost, imbalanced resolution transitions, and resolution mismatch. Applications of minimal error simulation methods to several complex high-Re flows reported in Section 4 provide evidence for this view [9,10,11]. The latter includes detailed analyses of remarkable computational cost advantages of minimal error simulation methods.
5. Major shifts of the use of computational simulation methods are driven by simple practical requirements and relative simplicity of alternatives. For example, the motivation of LES was the need to resolve flow; DES and WMLES developments (PANS and PITM methods are not very often applied) were driven by unaffordable LES computational cost requirements for high Re wall-bounded turbulent flows. So what may be the role of minimal error methods in the future? Apart from disappointment in looking for appropriate simulation settings of DES and WMLES methods, the strongest motivation for using minimal error methods can be the need for reliable predictions of very high Re turbulent flows, i.e., conditions where other computational methods are known to fail.

## Figures and Tables

**Table 1:** Summary of hybridization options.

O1. **Exact hybridization:** Exact hybridization results for $\psi_{\alpha }$ are: • no ϵ-eq.: $\psi_{\alpha }=P/\epsilon -\tau_{+}\left(P_{tot}/\epsilon_{tot}-1\right)\;\;\to \;\;Dk/Dt=k_{+}\left(P_{tot}-\epsilon_{tot}\right)+D_{k}^{*}$ • ϵ-eq.: $\psi_{\alpha }=\alpha -\tau_{+}\left(\alpha -1\right)\;\;\;\;\;\;\;\;\;\;\;\;\to \;\;Dk/Dt=P-\alpha \epsilon +k_{+}\left(\alpha \epsilon_{tot}-\epsilon_{tot}\right)+D_{k}^{*}$ This needs to consider more complicated equations ($\nu_{t}$ is replaced by $\nu_{t,tot}$ in $D_{k}^{*}$ and $D_{\epsilon }^{*}$). O2. **Reduced exact hybridization:** Otherwise, the neglect of Dk/Dt in regard to calculating $\psi_{\alpha }$ was found to be fully justified by applications. By using this assumption, we found • no ϵ-eq.: $\psi_{\alpha }=P/\epsilon -L_{+}^{2}\left(P_{tot}/\epsilon_{tot}-1\right)\;\;\to \;\;Dk/Dt=L_{+}^{2}\epsilon_{+}\left(P_{tot}-\epsilon_{tot}\right)+D_{k}$ • ϵ-eq.: $\psi_{\alpha }=\alpha -L_{+}^{2}\left(\alpha -1\right)\;\;\;\;\;\;\;\;\;\;\;\;\to \;\;Dk/Dt=P-\alpha \epsilon +L_{+}^{2}\epsilon_{+}\left(\alpha \epsilon_{tot}-\epsilon_{tot}\right)+D_{k}$ O3. **Approximated hybridization:** The neglect of Dk/Dt in regard to calculating $\psi_{\alpha }$ in conjunction with the RANS setting $\epsilon =\epsilon_{tot}$ in Equation (2) implies (see Appendix A) • no ϵ-eq.: $\psi_{\alpha }=P/\epsilon_{tot}-k_{+}^{3}\left(P_{tot}/\epsilon_{tot}-1\right)\;\;\to \;\;Dk/Dt=k_{+}^{3}\left(P_{tot}-\epsilon_{tot}\right)+D_{k}$ The latter specifies the O2 result for $\epsilon =\epsilon_{tot}$ considered ($L_{+}$ reduces to $k_{+}^{3/2}$).

## Appendix A. Approximated Hybridization

The involvement of a scale equation (for ϵ) significantly contributes to simplifying the structure of hybrid equations (via the replacement of the production term in the hybridization parameter). However, there are several usually applied codes that do not involve dissipation transport equations. In this case, algebraic (RANS-type) models for length scales are used to determine ϵ. The only consistent way to deal with this situation is to assume that $\epsilon =\epsilon_{tot}$. Fortunately, this approximation is usually a good assumption except close to walls. Correspondingly, a modification of the approach considered (leading to the O3 formulas in Table 1) is the replacement of ϵ in Equation (2) by the RANS-type $\epsilon_{tot}$ (including the corresponding replacement of ϵ in $D_{k}$) with the understanding that $\epsilon_{tot}$ can be provided:

(A1a) $$\begin{array}{c} \frac{Dk}{Dt}=P-\psi_{\alpha }\epsilon_{tot}+D_{k},\;\mathrm{where} \end{array}$$

(A1b) $$\begin{array}{c} \delta \left[\frac{D_{k}}{k^{3}\epsilon_{tot}}\right]=\frac{D_{k}}{k^{3}\epsilon_{tot}}\left(\frac{\delta D_{k}}{D_{k}}-3\frac{\delta k}{k}\right)=0. \end{array}$$

The relevant variation of $D_{k}$ arising from the structure of $D_{k}$ is involved in Equation (A1b). The normalized hybridization error λ reads, then (again, without involvement of Dk/Dt),

(A2) $$\frac{\lambda }{k^{3}\epsilon_{tot}}=\frac{P}{k^{3}\epsilon_{tot}}-\frac{\psi_{\alpha }}{k^{3}}+\frac{D_{k}}{k^{3}\epsilon_{tot}}.$$

The motivation for the normalization applied is the vanishing variation of the last term; see Equation (A1b). The requirement of a zero variation of the normalized error implies, then,

(A3) $$\delta \left[\frac{\psi_{\alpha }}{k^{3}}\right]=\delta \left[\frac{P}{k^{3}\epsilon_{tot}}\right].$$

We integrate this expression as before to obtain

(A4) $$\frac{\psi_{\alpha }}{k^{3}}-\frac{1}{k_{tot}^{3}}=\frac{P}{k^{3}\epsilon_{tot}}-\frac{P_{tot}}{k_{tot}^{3}\epsilon_{tot}},\;\mathrm{or},\;\psi_{\alpha }=\frac{P}{\epsilon_{tot}}-k_{+}^{3}\left(\frac{P_{tot}}{\epsilon_{tot}}-1\right),$$

where $k_{+}=k/k_{tot}$. It may be seen that this case agrees with Equation (18) for the case $\epsilon =\epsilon_{tot}$ considered because $L_{+}$ reduces to $k_{+}^{3/2}$.

The corresponding consequences for the hybridization mechanism can be seen by combining $Dk/Dt=P-\psi_{\alpha }\epsilon_{tot}+D_{k}$ with $\psi_{\alpha }=P/\epsilon_{tot}-k_{+}^{3}\left(P_{tot}/\epsilon_{tot}-1\right)$. We obtain

(A5) $$\frac{Dk}{Dt}=P-\left[\frac{P}{\epsilon_{tot}}-k_{+}^{3}\left(\frac{P_{tot}}{\epsilon_{tot}}-1\right)\right]\epsilon_{tot}+D_{k},\;\mathrm{or}\;\frac{Dk}{Dt}=k_{+}^{3}\left(P_{tot}-\epsilon_{tot}\right)+D_{k}.$$

It may be seen that this equation reflects the same hybridization mechanism as given in Equations (13) and (14). In regard to the required setting of $\epsilon_{tot}=k_{tot}^{3/2}/L_{tot}$, it is assumed that $L_{tot}$ is algebraically specified, and $k_{tot}$ can be obtained as described above. Required knowledge of $P_{tot}=\nu_{t,tot}S^{2}$ can be obtained correspondingly based on the definition of $\nu_{t,tot}=C_{\mu }k_{tot}^{2}/\epsilon_{tot}$.
